# Role of robotic surgery in general surgical procedures

**DOI:** 10.6026/973206300214076

**Published:** 2025-11-15

**Authors:** Ankit Ashok Agrawal, Pranavkumar Amrutlal Parthsarthi, Harsh Arvindbhai Patel, Kischentaran Ravindra Sanmugam, Arun H Vyas, Joyal James

**Affiliations:** 1 Department of General Surgery, Government Medical College Nagpur 440003, India; 2 Department of General Surgery, Nootan Medical College and Research Centre, Sankalchand Patel Vidyadham, Visnagar-384315 Gujarat, India; 3 Department of General Surgery, Dr. N D Desai Faculty of Medical Science and Research, Dharmsinh Desai University, Nadiad, Gujarat, Pincode - 387001, India; 4 Department of Trauma and Orthopaedics, Ninewells Hospital & Medical School, Dundee, United Kingdom; 5 Department of Anaesthesia, Dr. N. D. Desai Faculty of Medical Science and Research, Dharmsinh Desai University College Road, Nadiad, Kheda, Gujarat 387001, India; 6 Department of General Medicine, Medical University of Varna, Varna, Bulgaria

**Keywords:** Robotic surgery, general surgery, minimally invasive surgery, da vinci robot, surgical innovation

## Abstract

Robotic surgery has transformed the landscape of general surgical procedures by offering enhanced precision, visualization and control. This technology facilitates minimally invasive techniques in complex procedures, resulting in reduced recovery time and postoperative complications. Despite the high cost and technical demands, robotic-assisted surgery continues to expand in indications. Early evidence suggests improved outcomes in colorectal, hernia and hepatobiliary surgeries. Future integration with AI and improved cost-effectiveness may increase accessibility.

## Background:

Robotic surgery has arguably become the most important advancement in the history of minimally invasive surgery. In many ways, robotic surgery began with the introduction of robotic systems to extend the usefulness of the limitations of conventional laparoscopy and to improve dexterity, precision and visualization for surgeons to perform complex surgical procedures [1]. Since the da Vinci Surgical System was approved by the U.S.-Food and Drug Administration in 2000, robotic-assisted surgery has seen rapid proliferation across the globe [2]. Robotic assisted surgery provides advanced features to the operative environment such as three-dimensional high-definition magnification, wristed instruments with seven degrees of freedom, motion scaling and tremor filtration - allowing surgeons to see and control more advanced and complex tasks while maintaining a level of ergonomic comfort [3]. In general surgery, robotic platforms are used with increased frequency in diverse operations such as cholecystectomy, colorectal resection, hernia repair and procedures in the hepatobiliary arena. Evidence from a lot of clinical studies has reported that robotic assisted approaches have considerable advantages as compared to traditional open or laparoscopic surgeries [4]. Reports of major advantages have included decreases in intraoperative blood loss and postoperative pain scores, faster mobilization, decreased length of stay and fewer complications [5]. In addition, the ergonomic control features of the robotic systems have resulted in less risk of fatigue for the surgeons and the opportunity for advanced variation in education with simulation and training opportunities. However, there are barriers to broader acceptance [6]. The financial and maintenance burden, longer operating times during the early learning curve and need for specialized training are key obstacles to access, especially in low-resource healthcare settings [7]. Furthermore, the response for a more rigorous randomized controlled trial with long-term follow-up is needed in order to demonstrate robotic surgical assistance to be better in terms of cost-effectiveness and patient outcomes [8]. Therefore, it is of interest to compare the outcomes of robotic-assisted surgery and traditional surgical techniques in general surgical procedures.

## Materials and Methods:

The study was an observational study that took place over an 18-month period at a tertiary care educational hospital. We enrolled 120 patients who underwent general surgical procedures from the time they contacted our office until the end of the 18-month study after they met the eligible criteria. The patients were divided into two groups, those who underwent robotic-assisted surgery and those who underwent conventional surgery, which included laparoscopic and open surgery. Assignment to either group was driven by clinical need, patient preference and availability of the robotic surgical system at the time of surgery. All patients underwent a preoperative assessment which included a review of their pertinent medical history, physical examination, blood work and imaging studies from recent years as needed to determine surgical fitness and operational strategy. Both consolidated and robotic assisted surgeries were performed by experienced surgeons that had been appropriately trained in the technique of either and in either case, a standardized operative protocol was followed. Perioperative outcomes collected included intraoperative, patients' perioperative recoveries (including pain score, time to ambulation, length of stay and complications). Postoperative pain was assessed on the first postoperative day with a numerical rating scale. Postoperatively, we followed patients during their length of stay and their outpatient visits to county complications, readmissions, notifications, or milestones/markers of recovery.

Data were systematically recorded and analyzed statistically using SPSS software version 26. Continuous variables were expressed as the mean ± standard deviation and categorical variables were expressed as frequency and percentage. Descriptive statistics were used to compare the robotic vs conventional groups with a significance level defined as <0.05.

## Results:

A total of 120 patients were included in the analysis with 60 patients assigned to robotic-assisted surgery and 60 patients assigned to conventional surgery therapy. The two groups were similar in terms of baseline demographics and clinical characteristics, minimizing selection bias and allowing for valid comparisons. Intraoperative outcomes showed less bleeding associated with robotic cases but operative times were longer than conventional approaches. Post-operative recovery parameters favoured the robotic group with significantly less pain, ambulation sooner post-operative and shorter length of hospital stay than the conventional group. Complications were lower in robotic cases, as there were fewer incidences of wound infections and seromas. Subgroup analyses of individual procedures also demonstrated patterns of better recovery and less morbidity associated with robotic surgeries, such as hernia repairs and colectomies. At follow-up, patients who had robotic surgery returned to normal activities quicker and were less likely to require re-admissions after conventional laparoscopic surgeries, thus supporting the immediate postoperative advantages seen with robotic surgery. Overall, despite longer operative times with the robotic techniques, surgical recovery and decrease in complications were superior to standard approaches. Table 1 (see PDF) shows a comparison of operative time and intraoperative blood loss between robotic and conventional surgery. Table 2 (see PDF) depicts hospital stay, pain score on postoperative day one and time to ambulation for robotic and conventional surgery. Table 3 (see PDF) shows postoperative complications including wound infection, seroma and readmission rates. Table 4 (see PDF) depicts the distribution of common general surgical procedures performed with robotic and conventional approaches. Table 5 (see PDF) shows patient distribution based on age, sex, body mass index (BMI) and comorbidities. Table 6 (see PDF) shows operative and recovery outcomes stratified by procedure type. Table 7 (see PDF) differentiates complications into intraoperative versus postoperative and minor versus major. Table 8 (see PDF) shows postoperative follow-up parameters including time to return to normal activity, duration of readmission and reoperation rates. Table 1 (see PDF) demonstrates reduced blood loss but longer operative times with robotic surgery. Table 2 (see PDF) indicates significantly better recovery outcomes in robotic cases. Table 3 (see PDF) shows fewer complications in the robotic group. Table 4 (see PDF) confirms that procedure distribution was balanced. Table 5 (see PDF) establishes that baseline demographics were comparable between groups. Table 6 (see PDF) illustrates that across specific procedures, robotic approaches consistently resulted in less blood loss, reduced pain and shorter hospital stay. Table 7 (see PDF) provides a breakdown of complication types, showing fewer major events in robotic cases. Table 8 (see PDF) highlights favorable follow-up outcomes for robotic surgery, with earlier return to normal activity, shorter readmissions and lower reoperation rates.

## Discussion:

Robotic surgery presents numerous benefits over traditional surgery as it enables superior three-dimensional visualization, accuracy of instrument manoeuvrability and expanded range of motion to complete complex minimally invasive procedures [1]. Robotic-assisted surgery produced evidence of clear perioperative advantages over conventional surgery in this study, including decreased intraoperative blood loss and improved short-term recovery time. This study is consistent with previous studies reporting robotic surgery comparisons to conventional surgical techniques, specifically in decreased postoperative pain, earlier mobility and shorter length of stay [2, 3]. Although the operative times were longer in the robotic group, this did not negatively impact patient outcomes. Increases in operative time may be attributed to setup time, docking time and the learning curve with robotic systems. These benefits related to ergonomics decreased surgeon fatigue and improved precision, which may be related to the differences in intraoperative complications in the study [4, 5]. Systematic assessments on robotic surgery have demonstrated the ergonomic benefits associated with improved instrument articulation and motion scaling leads to improved safety with less complication during surgical procedures [6]. Cost is one of the main obstacles to the implementation of robotic-assisted surgical procedures. Robotic-assisted procedures have increased costs related to equipment, maintenance and increased operating room time in the learning phase of the procedure [7]. However, the possible impacts on long-term economy, through shorter hospital stays, fewer complications after surgery, less analgesic use and a quicker return to normal activity, may indeed equal or exceed the initial investment on certain procedures [8]. More in-depth cost-effectiveness analysis is warranted, particularly in healthcare systems where resources are at a premium. Robotic surgery had been particularly useful in anatomically complex operations like colorectal, hernia, foregut and hepatobiliary surgery, where precision is needed for better outcomes [9, 10]. This was seen in our subgroup analysis, in which patients undergoing robotic colectomy and liver resections had decreased postoperative morbidity and returned to normal activities sooner. The robotic systems allow for good and stable dexterity to perform precision dissections in a confined space [11]. Furthermore, the advancement of simulation training, virtual reality and standardized credentialing pathways to learn, teach and safely perform robotic surgery in clinical practice had a tremendous impact [12, 13-14]. Technological advancements are also continuing to facilitate innovation of robotic platforms with augmented imaging, Artificial Intelligence assistance and more advanced haptic feedback systems, which may improve surgical outcomes in the near future [15, 16]. Some of the primary strengths of our study consist of a balanced cohort with similar baseline characteristics and standardized surgical protocols conducted by experienced surgeons. However, some limitations must be considered. This was a single-center observational study and may be subject to selection bias. Operative times may decrease with time as surgical teams adapt to the learning curve related to robotic systems. In addition, the study was limited to short-term outcomes and long-term follow up, including recurrence rates, functional outcomes and quality of life, is required to fully evaluate the potential benefits of robot-assisted surgery.

## Conclusion:

Robotic surgery is a transformative modality in general surgical practice with clear benefits in selected procedures. Thus, on-going technological evolution and evidence-based integration will expand its role further despite costing.
